# Drug Resistance to Molecular Targeted Therapy and Its Consequences for Treatment Decisions in Non-Small-Cell Lung Cancer

**DOI:** 10.3389/fonc.2014.00190

**Published:** 2014-07-23

**Authors:** Johanna N. Spaans, Glenwood D. Goss

**Affiliations:** ^1^Ottawa Hospital Research Institute, Ottawa, ON, Canada; ^2^Ottawa Hospital Cancer Centre, Ottawa, ON, Canada; ^3^Department of Medicine, University of Ottawa, Ottawa, ON, Canada

**Keywords:** EGFR, inhibition, primary resistance, acquired resistance, molecular biology

## Abstract

Our ability to detect and directly target the oncogenic alterations responsible for tumor proliferation has contributed significantly to the management of lung cancer in the last decade. The therapeutic efficacy of molecularly targeted therapy is, however, mainly limited to patients harboring certain genetic mutations and is generally short-lived. Herein, we review primary and secondary drug resistance using the most well-studied of the molecularly targeted agents, the tyrosine kinase inhibitors targeting the epidermal growth factor (EGF) receptor, and the anaplastic lymphoma kinase (ALK) rearrangement, the current limitations of targeted therapies and their consequences on the management of patients with lung cancer.

The treatment of advanced non-small-cell lung cancer (NSCLC) had reached a therapeutic plateau prior to the introduction of molecularly targeted agents (MTAs), with a median survival of 8–12 months (1, 2). With an improved understanding of the molecular biology of lung cancer, enabled by advances in high-throughput technology, have come molecular therapies that target specific receptors and oncogenic pathways responsible for tumor growth and proliferation. Despite the demonstrated superiority of these MTAs over standard chemotherapy in subgroups of patients (3, 4), their therapeutic efficacy is limited to patients harboring the targeted genetic aberration and is generally short-lived. Any future advances in the survival of patients with advanced NSCLC will hinge on our ability to expand on the percentage of patients eligible and responsive to targeted therapy and our capacity to mitigate the mechanisms of acquired resistance that prevent long-term disease control.

As the most well-studied of the MTAs, tyrosine kinase inhibitors in NSCLC targeting the epidermal growth factor (EGF) receptor (erlotonib, gefitinib, and afatinib) and the anaplastic lymphoma kinase (ALK) rearrangement (crizotinib) provide a useful framework in which to understand the current limitations of molecularly targeted therapy and their consequences in the management of patients with NSCLC.

## Primary Resistance to EGFR Inhibitors

The EGFR pathway is known to be active in NSCLC (5) and protein overexpression is known to be associated with poorer prognosis (6). Early on in the clinical development of EGFR-tyrosine kinase inhibitors (EGFR-TKIs), which targeted this pathway, it was realized that patients whose tumor harbored an activating mutation in the EGFR gene at exons 19 and 21 had more dramatic responses and better clinical outcomes than their EGFR wild-type (W/T) counterparts (7, 8). This has resulted in some countries limiting regulatory approval in the first-line setting to patients whose tumors harbor these sensitizing mutations (9). Although common among lung cancer patients of Asian descent (10), sensitizing EGFR mutations are relatively uncommon in North American and European NSCLC populations with a prevalence of ~15% in patients with advanced non-squamous histology (11). Further, despite their heightened sensitivity to EGFR-TKIs, as many as one third of NSCLC patients with tumors with sensitizing EGFR mutations do not respond to targeted therapy (12, 13).

The mechanisms of primary resistance to EGFR-TKIs are best considered in terms of patients with tumors with (EGFR mutant) and without (EGFR W/T) sensitizing mutations. In the latter case, patients may not respond to EGFR-TKIs because their tumors are being driven by other oncogenic pathways that are not sensitive to EGFR inhibition. Indeed, different oncogenic alterations have been identified in up to half of patients with EGFR W/T disease (14). Importantly, the successful targeting of one such alteration, namely the ALK gene rearrangement with crizotinib (15) demonstrates the feasibility of addressing this form of primary resistance in the EGFR W/T population. Therapies targeting other mutations that commonly occur among EGFR W/T patients such as c-ros oncogene 1 (ROS1), ret proto-oncogene (RET), v-raf murine sarcoma viral oncogene homolog B (BRAF), and the human EGF 2 (HER2) are currently under development.

In addition, primary resistance in the EGFR W/T population may be the result of activation of alternate parallel signaling pathways, which can overcome EGFR blockade, that are independent of a pathway-specific activating mutation. Activation of the insulin-like growth factor receptor (IGFR) pathway (16) is one such potential mechanisms of primary resistance. Blockade of these alternate pathways to enhance EGFR TKI efficacy is a strategy that is being investigated, but to date has yielded mixed results (17–19). Lack of response to EGFR-TKIs among patients with W/T tumors may also be due to incomplete binding of the drug to the EGF receptor or because of insufficient drug concentration necessary for effective pathway blockade (20). Further, as only one of four receptor tyrosine kinases in the ERbB family (21, 22), the isolated targeting of the EGF/Erb1 receptor may not prevent auto-phosphorylation and downstream pathway activation by the other receptors (i.e., Erb2, Erb3, Erb4). Newer second-generation irreversible pan-HER tyrosine kinase inhibitors such as dacomitinib and afatinib that target multiple receptors are being evaluated in both EGFR W/T and EGFR-mutant populations as a strategy to enhance and prolong treatment response (23, 24).

In patients with tumors with EGFR sensitizing mutations a number of factors have been identified in the primary resistance setting, which may modulate or blunt the therapeutic efficacy of EGFR-TKIs. While most oncogenic driver mutations are mutually exclusive in context of lung cancer, the co-existence of EGFR mutations with other oncogenic alterations, including class A phosphoinositide 3-kinase (PI3KCA), have been reported (25). Activation of compensatory signaling pathways by these other mutations may afford continued disease progression and negate or circumvent clinical benefit derived from EGFR TKIs in patients whose tumors harbor EGFR mutations. The dual targeting of co-existing mutations with combination therapy in EGFR-mutant disease is currently under investigation (clinical trials.gov: NCT01570296).

In a similar fashion, exogenous factors, including MED12-mediated transforming growth factor beta (TGF-β) activation (26) and hepatocyte growth factor (HGF) ligand overexpression (27), may enable the activation of alternate signaling pathways in tumors that may override the pathways inhibited by the EGFR-TKIs. The inhibition of these compensatory pathways in tumors with EGFR sensitizing mutations is a hot topic in clinical research with the testing of a number of combination therapies that include agents to overcome TGF-β and HGF-mediated resistance [e.g., heat shock protein (Hsp) 90 inhibitors] in both the primary (clinicaltrials.gov: NCT01714037) and resistance setting (clinicaltrials.gov: NCT01259089, NCT01288430, NCT01851096).

Finally, exogenous apoptotic factors have been identified that may modulate the impact of EGFR TKIs in patients with tumors with sensitizing EGFR mutations and may explain their variable treatment response. Increasing evidence suggests that expression levels of proapototic BH-3 only molecule (BIM) can influence treatment-induced apoptosis (28, 29) and further that pre-treatment BIM expression may play a role in treatment response to many kinase inhibitors across many disease sites (30, 31). While in lung cancer, it has been shown that low pre-treatment BIM levels are associated with shorter time to progression (29), available pro-apoptotic assays are not currently being used in clinical practice to predict treatment response. Targeted therapies with B-cell lymphoma 2 (blc2) inhibitors that enhance apoptosis, however, are currently being evaluated in combination with EGFR-TKIs as a strategy to enhance treatment response (clinicaltrials.gov: NCT00988169).

Despite early and dramatic treatment responses in up to two thirds of patients with EGFR sensitizing mutations, most patients will eventually progress while on therapy within a year of treatment initiation (12). Beyond the level of pre-treatment apoptotic factors, such as BIM discussed previously, a number of other factors have been suggested to influence the development of clonal and sub-clonal EGFR-resistant cell populations. Specifically, both factors affecting the drug metabolism and characteristics of the treatment schedules may impact the development of acquired resistance in previously responsive patients (32). While the higher metabolic clearance of EGFR-TKIs among smokers and fast metabolizers has long been recognized as a negative predictor for time to progression (33), only recently have the pharmacokinetics of different dosing schedules been considered for their potential influence on the evolution of drug resistance to EGFR TKIs. Specifically, based on evolutionary modeling and clinical data, it has been proposed that pulsed high dose with continuous low dose EGFR-TKI treatment helps to maintain sensitive cell populations and may extend the therapeutic benefit of EGFR-TKI therapy beyond progression (34). While standard once daily dosing continues to be used in the clinic for approved MTAs, research is on-going to define characteristics of the treatment regimen that may delay disease progression and optimize therapeutic outcomes with EGFR-TKI therapy (clinicaltrials.gov: NCT01967095).

Although criteria for acquired resistance have now been developed (35), resistant disease is best considered along an evolutionary continuum, where resistant clones eventually overrun EGFR-sensitive cells, leading to the clinical characteristics of disease progression. The existence of EGFR-sensitive cells in tumors that progress is supported by reports of clinical response in patients re-challenged with EGFR-TKIs (36) and also by reports of disease flare in up to 15% of patients who are taken off EGFR-TKI therapy at disease progression (37).

## Secondary EGFR-TKI Resistance

Genetic adaptations and altered network signaling pathways invariably lead to drug resistance in patients whose tumors harbor EGFR sensitizing mutations who initially respond to EGFR-targeted therapy (acquired resistance). Molecular profiling of tumors with acquired resistance to EGFR-TKIs has identified a number of resistance mechanisms and dominant acquired resistance phenotypes, which may be useful in guiding future treatment. The most common mechanism of acquired resistance to EGFR-TKI is the development of a second mutation of the EGFR that is resistant to therapy. While a number of secondary mutations have been identified (38), the most common “gatekeeper” mutation is that of the T790M, which occurs in 50–60% of patients with acquired resistance to EGFR-TKIs (39). This secondary mutation is believed to exert its effect by enhancing ATP kinase affinity, thereby decreasing sensitivity to the ATP-competitive EGFR TKIs (40). Importantly, the development of secondary resistance mutations in the EGFR kinase domain has implications in the re-challenging of patients with previously sensitive disease and has fueled research in the development of second and third generation inhibitors (41–45). Despite encouraging phase II data of one such second-generation inhibitor (dacomitinib) in previously treated patients (41, 42), emerging phase III data suggests that there is no overall survival benefit associated with its use in previously treated EGFR W/T patients or those with acquired EGFR-TKI resistance (23). Similarly, while interim analysis of another second-generation irreversible ErbB family blocker (Afatinib) in patients with acquired resistance to EGFR-TKIs suggested improved progression-free survival (PFS) (43), the lack to an overall survival benefit observed in this phase 2b/3 randomized trial (24) does not support the strategy of extended EGFR blockade in the EGFR-resistant population.

Most recently, the finding of a T790M mutation in tumors at the time of initial diagnosis (44, 45) has implicated the mutation in primary EGFR-TKI resistance, suggesting that up-front treatment with second/third generation EGFR-TKIs may confer added benefit over their use in the second-line setting in patients with T790M-mediated acquired resistance. Indeed, preliminary clinical reports of second-generation EGFR inhibitors in the first-line treatment setting support their up-front use in patients whose tumors harbor EGFR mutations (46, 47).

An alternate mechanism of secondary resistance is the activation of other signaling pathways by adaptive *de novo* alterations that develop outside the EGFR kinase domain in response to treatment. A number of these alterations have been identified; the most well-studied being MET amplification, which occurs in 10–20% of patients with EGFR-TKI resistant disease (48). Other less common mutations include HER2 amplification (49, 50), activation of PIK3Ca (51) and BRAF (52), and loss of phosphatase and tensin homolog (PTEN) function (53).

Crosstalk between key signaling pathways may also play a role in the development of acquired resistance to EGFR inhibitors. Specifically, the activity of the angiogenic VEGF pathway has been suggested to play a role in resistance to EGFR-TKIs (54), which is not surprisingly given the common downstream effectors shared by these parallel pathways (55). Although preclinical data across different tumor types (56, 57) and early phase lung cancer clinical trials (58, 59) pointed to the potential utility of dual inhibition of VEGF and EGFR, phase III data of the dual inhibitor vandetanib suggest that this is not a promising approach to overcome acquired resistance to EGFR-TKIs in advanced NSCLC (60).

Finally, a less common but well documented mechanism of acquired resistance to EGFR-TKIs is histological transformation from NSCLC to SCLC or epithelial–mesenchymal transition (EMT), which has been reported in up to 3% of EGFR-TKI resistant patients (61). Increasing evidence suggest that these transformations are linked to the activation of the AXL kinase, the inhibition of which may restore EGFR-TKI sensitivity in previously resistant cells (62). Collectively, these mechanisms clearly demonstrate the multitude of adaptive strategies developed by the tumor to ensure its continued growth and underscores the complexity of treating EGFR-TKI-resistance disease.

While the above resistant disease phenotypes are useful in the classification of acquired resistance, these adaptive mechanisms may not be mutually exclusive. Indeed, it has recently been proposed that T790M mutations and MET amplifications are complementary and may co-exist in the development of drug resistance (63). In addition, oncogenic driver mutations may be tumor specific, as different driver mutations from different tumor sites within the same individual have been identified in patients with EGFR-TKI resistant disease (39), further illustrating the challenges in managing patients with acquired resistance.

## Primary Resistance to ALK Inhibitors

Between 1 and 3% of patients with advanced NSCLC have tumors that harbor sensitizing chromosomal rearrangements of the ALK gene (64–66). The ALK inhibitor crizotinib has recently been approved for the treatment of patients with ALK-positive tumors, however, as with EGFR-TKIs not all patients respond to therapy. Specifically, while phase III studies have shown that crizotinib improves PFS compared to chemotherapy in previously treated NSCLC patients with ALK-positive disease (HR = 0.49 95% CI: 0.37–0.64, *p* < 0.001), only 65% of patients were shown to respond to therapy (67). While primary resistance among patients with ALK-positive tumors is less well-understood, the occurrence of drug-resistant ALK mutations and compensatory mechanisms have been advanced as potential mechanisms of primary resistance (68).

In summary, less than 20% of patients have tumors with an EGFR or ALK mutation at the time of diagnosis, and of these, only 60–70% of patients respond to currently available MTAs. Therefore, we are mandated to address the approximately 80% of patients whose tumors are *de novo* resistant to EGFR and ALK inhibition, a percentage of whom are resistant due to other oncogenic driver mutations such as Kirsten rat sarcoma viral oncogene homolog (KRAS), BRAF, and RET, among others. Many investigations targeting these mutations are on-going.

## Secondary Resistance to ALK Inhibitors

While secondary mutations in the ALK domain have been identified in approximately one-third of the patients with acquired resistance to ALK inhibitors (69), unlike acquired resistance to EGFR-TKIs, there does not appear to be a dominant secondary mutation. Further complicating the management of such patients, multiple mutations within the same individual have also been reported in patients with acquired resistance (70). Of note, second-generation ALK inhibitors have, however, recently shown high response rates (48% confirmed responses) in patients previously treated with crizotinib, in tumors with and without secondary mutations in ALK (71). These results support the importance of ALK in crizotinib-resistant disease and the continued effort in targeting the ALK domain.

## Treatment Implications

Since the publication of the initial reports over a decade ago, we now have a much better understanding of which patients stand to benefit most from targeted therapies with EGFR and ALK tyrosine kinase inhibitors and the intrinsic and adaptive mechanisms that limit treatment response and inevitably lead to acquired drug resistance. Despite these treatment advances, there are currently a limited number of therapeutic options available to patients not harboring sensitizing EGFR mutations or rearrangement of the ALK gene. Further, the mechanisms of acquired resistance among those who initially respond to treatment remain uncharacterized in almost 40% of patients with acquired resistance (72). That said, many agents targeting other oncogenic mutations are in phase III development, and in the near future will expand the armamentarium of targeted therapies available in the treatment of advanced NSCLC. The following strategies are proposed in the current and future management of patients with NSCLC.

All patients presenting with advanced NSCLC should be screened for all known oncogenic driver mutations with treatment assigned accordingly based on available molecularly targeted therapies. With the approval of second line and third line (T790M specific) EGFR-TKIs, it may also be useful to screen up-front for T790M mutations and preferentially treat patients harboring these mutations with these second and third generation therapies, given the shorter PFS that patients harboring this mutation experience with first-line reversible EGFR-TKIs (44). In addition, for patients harboring sensitizing EGFR mutations, the assessment of pre-treatment BIM expression may be a useful approach to help to optimize EGFR-TKI treatment outcomes, with the addition of anti-apoptotic inhibitors such as Blc2 to the treatment regimen.

While alternate dosing schedules, such as pulsed high dose with continuous low dose may be shown to delay time to disease progression, current treatment regimens of approved targeted agents are limited to once daily dosing. EGFR-TKI treatment should ideally be continued in the case of disease progression until the initiation of second-line therapy, given the potential for disease flare (37) and data that suggest that patients may benefit from continued treatment beyond progression (73, 74). As it has been shown that isolated sites of disease progression may be successfully treated while continuing on EGFR-TKIs (75), the decision to discontinue EGFR-TKI therapy at disease progression should be considered in the context of available therapeutic alternatives and the potential benefit of continued EGFR-TKI therapy. For example, treatment with afatinib in addition to chemotherapy has recently been shown to delay progression over chemotherapy alone (5.6 vs. 2.8 months) in patients who had progressed on afatinib (76).

The optimal treatment of patients with tumors that harbor EGFR mutations and ALK gene rearrangement who develop acquired resistance to EGFR and ALK tyrosine kinase inhibitors has yet to be defined. While most patients are managed with chemotherapy, the evidence to support this therapeutic approach is limited and the documented response rate with chemotherapy in patients with EGFR-resistance disease is l0–20% (77). As more targeted therapies become available, a more informed approach to the treatment of acquired disease to targeted therapies may emerge through rebiopsy at the time of disease progression and tailoring of subsequent mechanism-based therapies.

Lung cancer is a heterogenous disease and resistance mechanisms to targeted molecular therapy are many. Given the multitude of signaling pathways and the evolving characteristics of resistant disease, an up-front combination therapy that simultaneously inhibits multiple resistance pathways is likely to yield better clinical outcomes. Personalized targeted therapy at the time of disease recurrence may further improve survival. Importantly, an aggressive front-line strategy and a tailored management approach in the case of resistant disease has been successfully employed in the management of other diseases, including HIV (78), which has now come to be considered a chronic disease. Whether advanced lung cancer may someday have a similar clinical outcome remains to be seen. To achieve this, attention must be directed at reducing the toxicity of combination therapies and greater efforts made to define the molecular basis of acquired resistance.

## Conflict of Interest Statement

The authors declare that the research was conducted in the absence of any commercial or financial relationships that could be construed as a potential conflict of interest.
